# Effectiveness of feedback-based technology on physical and cognitive abilities in the elderly

**DOI:** 10.3389/fnagi.2022.1050518

**Published:** 2022-11-10

**Authors:** Maria-Luisa Benitez-Lugo, Carmen Suárez-Serrano, Alejandro Galvao-Carmona, Manuel Vazquez-Marrufo, Gema Chamorro-Moriana

**Affiliations:** ^1^Department of Physiotherapy, University of Seville, Seville, Spain; ^2^Department of Psychology, University of Loyola, Seville, Spain; ^3^Department of Experimental Psychology, University of Seville, Seville, Spain

**Keywords:** ageing, balance, gait, attention deficit, exercise, feedback-based technology, prevention

## Abstract

Aging raises a social and health challenge because the passing of time causes changes in cognitive and physical functions that impair functionality and quality of life. In addition, advancements in technology and information technology have led to the development of new techniques for retraining based on the feedback technology provides. To solve the negative consequences of aging, a randomized clinical trial was carried out to assess the effectiveness of a protocol using feedback-based technology to improve physical and cognitive functions in older adults. For the purpose of this study, 200 patients were selected from a Social and Community Services Center in the province of Seville and only 46 of them became participants of the study (after applying the inclusion criteria). These patients were divided into two groups: control and experimental. Physical and cognitive abilities were assessed using the *Miniexamen cognoscitivo Test* (an adaptation of the MiniMental examination test), Yesevage’s Depression Scale, Oddball Test, Attention Network Test, Berg Scale, Tinetti Scale, and Timed Up and Go Test. The intervention applied to the experimental group consisted of a supervised protocol using the Nintendo^®^ Wii video game console and the Wii-Fit© video game during 16 sessions, 2 times a week with a duration of 30 min per session. The control group did not receive any treatment. The experimental group showed statistically significant improvements in all the physical variables (balance, gait, autonomy, and fall risk), as other authors had proven, and in memory and reaction times; there were no improvements in attention networks. The control group (placebo) even showed a decrease in their functions, with worse results on the Timed up and Go test Scale. The intervention using feedback-based technology has been proven effective in improving physical and cognitive abilities and in preventing and promoting healthy aging.

## Introduction

The progressive increase in life expectancy in Western Europe, Spain and Italy having the eldest population, creates more long-lived societies. The age group over 85 is the segment of the population that has grown the most and it is more susceptible to disease and disabilities as well. Taking action in this situation is a challenge for the sustainability of modern societies (Christensen et al., 2009).

Although the process of aging is a natural one, controlling certain external factors could optimize aging to the point of even preventing dependency (Panza et al., 2018). This process is related to alterations in some abilities including balance, gait, and attention.

The ability to control balance during basic activities of daily living (BADLs) is impaired as a consequence of the deterioration of the sensory systems, the cognitive system, and the musculoskeletal system. Consequently, many older adults can modify their gait and suffer a higher risk of falling during their daily activities. In most cases, these falls and their related injuries cause deterioration in the quality of life, and result in physical limitations, anxiety, loss of confidence, and fear of falling (Dunsky, 2019).

In addition, there are consequences in the nervous system such as the reduction of brain volume, slower synaptic transmission, and lower number of neurotransmitters. The prefrontal cortex is one of the most affected areas because of aging and it is essential for the development of executive functions, attentional networks, and the ability to be resilient during different situations in life (Zhao et al., 2018). In this sense, it is worth highlighting the connection between executive functions and gait (Yogev-Seligmann et al., 2008), because if there is an impairment in the prefrontal cortex, it not only has cognitive consequences but impairments in gait and postural control too; and therefore, it affects the independence of the elderly and increase the risk of falling (Blanchet et al., 2018). The deterioration of the prefrontal cortex plays a key role in cognitive decline and alterations of other cognitive functions. As a result, physical abilities can deteriorate too (Allain et al., 2007). Many cognitive capacities participate in balance and gait, however, attention plays a fundamental role and it suffers alterations during aging (Kilb and Naveh-Benjamin, 2007), although there are certain contradictions regarding how these attentional networks change over the years: some authors believe that the alerting network is the only one affected (Jennings et al., 2007; Gamboz et al., 2010), others affirm that the alerting and the executive networks are affected (Vazquez-Marrufo et al., 2011; Zhou et al., 2011); this could be caused by the influence aging has on the prefrontal cortex; on the dopaminergic system; on the different functions of specific genes and on the effect of the interaction of attention networks. Concerning these networks, the effect on the alerting network is large at the beginning of the evaluation with the Attention Network Test, until the orienting and executive networks became more efficient and alertness decreases (Fan et al., 2009; Vazquez-Marrufo et al., 2011).

Nevertheless, the studies that have analyzed attentional deterioration during aging have used instruments with different conditions –in the number of essays and the presence of any cues or blocking analysis. This can be seen in the review by Mcdonough et al. (2019), in which they affirm that impairment in the executive network –which is usually compensated in healthy aging– is not compensated in pathological aging. For this reason, the behavioral information the attention network provides could be used as a tool to predict whether there is pathology in the brain or not. Consequently, the correct effectiveness of the attention networks depends on balance, skill in gait, and the lack of fall risk –this last aspect is essential for the creation of programs focused on fall prevention, an idea suggested in social policies (Rodriguez-Laso et al., 2018).

Designing programs that combine balance and coordination exercises would challenge postural control systems in different ways. This could be similar to real situations that older adults could experience during their daily life activities. In addition, the combination of dual activities and functional challenges while maintaining balance stimulates mechanisms of sensory and neuromuscular control; these types of activities could interact in physical and cognitive ways (Dunsky, 2019). The improvement of technology and information technology has led to the development of new techniques that achieve gait retraining due to the feedback that technology provides. The main idea of feedback is the ability to voluntarily control and change different bodily functions or biological processes when information about them is given. Feedback gives specific information about an aspect the person is not aware of. These feedback-based treatments can use robot-assisted movement, virtual reality technology, or inertial monitoring devices that can use visual, acoustic, or haptic feedback (Chamorro-Moriana et al., 2018). Resources such as interactive games with virtual reality devices provide inherent feedback and a larger number of movement repetitions in each session. Additionally, it provides the opportunity of improving physical and cognitive abilities at the same time (Iruthayarajah et al., 2017).

In this sense, the application of technology based on feedback could be a tool that could make the patient have to anticipate, maintain and react, putting into action their motor, sensory and cognitive abilities, depending on the context and the demands of the task. For all this, the use of sensory integration may provide an impact on motor, cognitive and social aspects (Camarata et al., 2020).

After analyzing how aging influences motor and cognitive skills, given its relationship with the state of attentional networks, functionality and autonomy, it is necessary to frame our research in the use of information technology. In this sense, the reviews developed show studies that focus the benefits exclusively on cognitive functions or balance or on the prevention of falls in older people (Chao et al., 2015; Choi et al., 2017; Hill et al., 2017; Karasu et al., 2018; Cavalcante Neto et al., 2019; Marques-Sule et al., 2021), as well as in specific pathologies such as multiple sclerosis (Waliño-Paniagua et al., 2019) or cerebrovascular accidents (Cano-Mañas et al., 2020).

However, in none of the cases is an exhaustive evaluation of the participants designed to assess the state of the physical, cognitive and emotional areas, nor to holistically integrate a task using the new technology that involves all areas at the same time. This gear could have a three-dimensional effect at the level of neural plasticity. Likewise, proposing an interdisciplinary intervention with stimulation in the respective spheres of the participants could be considered preventive and protective of the effects derived from situations of dependency. In this sense, the summative effect of the task in the different areas, as well as the incorporation of the analysis of the effect of attentional networks in the process, can add an innovative approach to actions developed with the use of technology based on feedback.

For all these reasons, this research is developed that aims to analyze the effectiveness of technology based on feedback for the optimization of the physical, emotional and cognitive functionality of the elderly through a specific tool. The first hypothesis of this study is that doing the task protocol using the Nintendo^®^ Wii video game console improves the memory, mood and attention of older adults. The second hypothesis is that the protocol used in this trial improves balance and gait, and prevents falls in older adults.

## Materials and methods

The subjects of the study were blinded. The professionals who were A randomized clinical trial was designed, registered as NCT04615897, to assess the effectiveness of using a feedback-based technological device (Nintendo^®^ Wii video game console) on physical and cognitive abilities. The study was planned in accordance with the guidelines outlined in the Declaration of Helsinki (THE WORLD MEDICAL ASSOCIATION, INC. DECLARATION OF HELSINKI Ethical Principles for Medical Research Involving Human Subjects) and it received the approval of the local ethics committee of experimentation of the University of Seville (see *ethical considerations*). Informed Consent was signed by all participants. Recommendations of the SPIRIT declaration were followed for the protocol of the clinical trial (Chan et al., 2013). Oxford (Sousa et al., 2017) and Consort Statements (Cobos-Carbó and Augustovski, 2011) were considered for its development.

The subjects of the study were blinded. The professionals who were responsible for the evaluations were blinded. Physiotherapists that performed the therapy were not blinded.

### Participants

Participants were recruited from a Social Community Services center, located in the province of Seville. The inclusion criteria were: being over 60 years old and not having any visual or hearing impairment (or if they had any, using the required aid, for instance, glasses). The exclusion criteria were: having a cognitive impairment, having a musculoskeletal condition that would prevent the subject from doing the activity, having a score > 9 on the Geriatric Depression Scale, sleeping less than 6 h a day, taking medication that could interfere with balance or the cognitive area or being vulnerable to the device flash. Each phase of the research is represented in a flowchart designed following the Consort Statements (see *Results*).

All the users carried out memory workshops (consisting of reading current news and commenting on it, puns and remembering images) and joint mobility workshops (consisting of carrying out active mobilizations of the different body joints) in their daily life in this center. This type of intervention is called usual care.

### Sample size

The sample size was determined by conducting a pilot study on 30 subjects (15 assigned to the experimental group and 15 assigned to the control group). Regarding the improvement in the gait variable between the control and experimental groups, an effect size of 1.41 was obtained. Later, the free software package “Gpower 3.1.3” for Windows was used. The data provided to the program for this calculation were: one-sided hypothesis, α error of 0.05 (95% confidence level), a study power of 80% and an effect size (d) of 1.41. Under these conditions, the estimated sample size is 28 subjects. Assuming a possible loss of 5% and applying the formula N = n/(1-%losses)2, a definitive sample size of 32 subjects is obtained. Finally, 48 (20 subjects in the control group and 28 subjects in the experimental group) are included, due to possible losses, possible complications derived from age and possible lack of adherence to treatment.

### Randomization

The randomization of the participants was conducted using opaque sealed envelopes, distributed into two groups, control and intervention, so that 28 envelopes were generated for the experimental group and 20 for the control according to the results of the sample size. In this study control group contains those participants with usual care (memory workshops and joint mobility workshops in the center) and experimental or intervention group: usual care (memory workshops and joint mobility workshops in the center) + sessions using the Nintendo^®^ Wii game console.

To maintain blinding, they were designated group 1 and group 2, rather than the control or experimental group, respectively.

### Trial variables

The study contains an independent variable: complete the task using feedback-based technology; five dependent variables: attention, memory, balance, gait, and autonomy and risk of falling, and difference variables. The study considers: gender, handedness, age, weight, years of education, depression, sleeping hours, presence of sensory deficits and their possible aids, and the stimulant tasks participants could perform as moderating or personal variables of the research. Below, trial variables, their function, characteristics, and level of measurement are described.

– Activity designed using feedback-based technology: participants complete a task using the Nintendo^®^ Wii video game console (detailed below). This variable divides the subjects into two categories: the ones that had had the treatment (had completed the task) and the ones that had not (had not completed the task). Therefore, it is a dichotomous nominal variable. It has been codified with the command “values” in the SPSS 18.0 computer program.

– Attention: obtained with two tests designed with the E-prime computer program. These tests were developed in the Psychophysiology laboratory of the University of Seville (Oddball Test and Attention Network Test-ELDERLY). The tests are constant quantitative measurements that provide the amount of precision (in percentages) and the reaction time (expressed in milliseconds).

– Memory: obtained with the *Miniexamen Cognoscitivo* scale (an adaptation of the Mini-Mental Examination test). Once the test is completed, the subject obtains a score that determines if there is a cognitive impairment (that would be a quantitative discrete variable).

– Balance: obtained with the use of the Berg Balance Scale and the Tinetti Test (only the balance section is used). This is considered a quantitative discrete variable.

– Gait: obtained with the Timed Up and Go Test. The time spent completing the test is a quantitative constant variable.

– Autonomy and risk of falls: obtained with the Tinetti test —a tool that assesses balance, gait, and risk of falls at the same time. It is considered a quantitative discrete variable.

– Difference variable: a set of dependent variables was added to the study, referred to as “difference variables”. These variables represent the differences between pre-test and post-test data in all the variables that were assessed at the beginning and the end: attention, memory, balance, gait and autonomy, and risk of falling. These variables have the same metric properties as the original variable, and they had been taken into account to facilitate statistical analysis of the data.

– Other variables to be controlled: a series of controlled modifying variables including gender (qualitative dichotomous nominal variable), the categories of which were male and female; weight (constant quantitative variable) measured in kilograms using a scale; age (constant quantitative variable) expressed in years; handedness (constant qualitative nominal variable) with three categories: right-handed, left-handed and ambidextrous; years of education (constant quantitative variable); hours of sleep (constant quantitative variable) measured in hours of sleep a day, and depression (quantitative discrete variable) measured using the Yesavage Depression Scale and that is included in the study because depression is related to decrease in attentional abilities, less implication during the experiment, an incidence in the target population and/or a reduction in the memory processes.

### Assessment tools

Different tools were used to quantify cognitive skills (memory, attention, and depression) and physical skills (balance, gait and autonomy, and risk of falls). Each one is explained below.

– Geriatric Depression Scale of Yesavage (Matias et al., 2016): the subject has to answer 15 yes or no questions. Each answer has a score of 0 or 1. The total sum provides the final result of the scale. A score from 0 to 5 means an absence of depression, a score from 6 to 9 suggests moderate depression and a score from 10 to 15 indicates severe depression.

– Oddball paradigm: chess boards (some were black and white, and others were red and white) appeared on the computer screen. There could be time differences in the appearance of these stimuli. The subject was asked to press the mouse button only when the white and red board appeared on the screen. The task consisted of 200 attempts and only 50 of them required a response from the subject. At the end of the task, the precision percentages and reaction time of each participant and each measurement were calculated.

– Attention Network Test-ELDERLY (Vazquez-Marrufo et al., 2011): using a computer, participants were asked to press either the left or right mouse button depending on the orientation of the target stimulus. In each trial, two stimuli were presented: the first one worked as a warning (no cue, center cue, and spatial cue) that anticipates the target stimulus (a group of arrows that can be congruent or incongruent). Subjects had to indicate the orientation of the center arrow. The test was divided into two blocks of 72 trials each (with a 5-min break between blocks to avoid fatigue in participants). Stimuli size was increased for better visualization. The angle view covered with the target stimuli had a width of 7.37° and a height of 0.86°. The warning cue had a width of 0.86° and a height of 0.86°. The cue stimulus had a duration of 150 ms and the target stimuli had a duration of 350 ms. The time passed between the cue and the imperative stimulus was 850 ms and the interval between the imperative stimulus and the next cue was variable with two levels: 1000 and 150 ms. The subject had a response time of 1000 ms after the appearance of the imperative stimulus. After completing the task, the effectiveness of the alerting network (subtracting the reaction time obtained for the stimuli preceded with the condition “no cue” and the ones preceded with the “central cue”), the orienting network (subtracting the essays started with a “central cue” and the ones started with a “spatial cue”) and the executive network (subtracting reaction times of the “incongruent stimuli” and the “congruent stimuli”) of every participant is obtained.

– Mini-Mental State Examination (MMSE): the Spanish version (*Miniexamen Cognoscitivo*) is a 30-question assessment grouped into 5 different sections that evaluate orientation; encoding memory; concentration and calculus; retrospective memory and language and production. The total score of the test is obtained with the sum of all the score sections. Having a score of <24 suggests dementia. The closer the score is to 30, the more optimal cognitive abilities are.

– Berg Balance Scale (Downs, 2015): the overall score provides an estimation of the general state of balance. It informs if there is a dependence (the need for a wheelchair), a need for aid or there is total dependence on walking. The patient has to perform 14 items with a score from 0 to 4. The total value is calculated by adding up the scores. Having a score from 41 to 56 means a good balance, from 21 to 40 indicates certain difficulties regarding balance and from 0 to 20, balance is impaired.

– Timed Up and Go test (Browne and Nair, 2019): in which the subjects are required to stand up from a chair, walk 3 meters and return to the starting point. If the test is completed in less than 10 s, it is considered a normal gait; if it is completed in 11 to 20 s, there is a moderate risk of falls and if it is completed in more than 20 s, there is a high risk of falls.

– Tinetti Test (Park, 2018): it assesses balance and gait with a total score of 28 points. Balance is calculated with 9 points, with a maximum score of 16. Gait is calculated with 7 points, with a maximum score of 12. It also assesses the risk of falls and autonomy. A score of <19 predicts a high risk of falls; a score between 19 and 23 predicts a moderate risk of falls and a score between 24 and 28 predicts a low risk of falls.

### Materials used for the task using feedback-based technology

**FIGURE 1:**
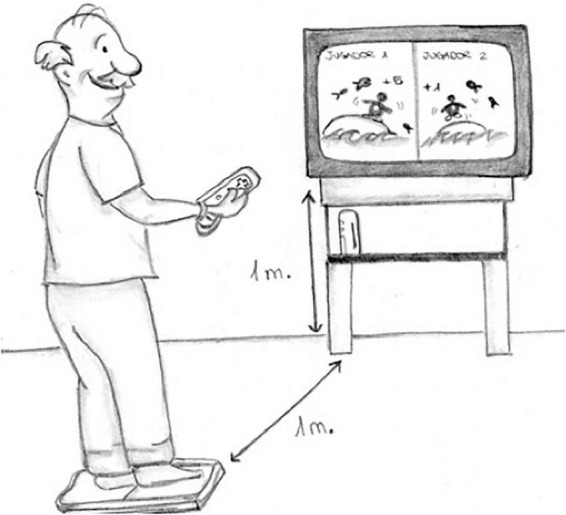
Distances marked on the task.

– Nintendo^®^ Wii video game console: this device is used with a long remote with a few buttons that works with gyroscopic technology which enables the remote to detect movement, distance, angles, and speed (if it is combined with the wireless device placed on top of the monitor). This device has a speaker that provides feedback to the experiment. The main technical characteristics of the video game console are: processor (IBM Brodway 729 MHz), storage (512 MB Flash memory card, SD and SDHC cards, and Nintendo GameCube memory card), Graphics (ATI Hollywood 243 MHz), and Connectivity (Wi-Fi, Bluetooth 2.0, 2 x USB 2.0, LAN adaptor via USB 2.0).

– Game used for the experiment (Wii-Fit(©): this game is used with the platform “Wii Balance Board” (which detects pressure). The four pressure sensors are connected to a wireless remote and to the sensor bar placed above the monitor. Once users had registered, specific characteristics can be assessed and they can get access to four types of exercises: yoga, strength training, balance, and aerobics. The proposal for this clinical trial was the video game “Penguin Slide” (found in the “balance” category) and the video game “Step Plus” (found in the “Aerobics” category). For the purpose of this activity, the “Wii Balance Board” was placed one meter away from the monitor, which was placed on a table one meter above the floor; it can be seen in Figure 1. The first game is aimed at the transfer of weight to both hemibodies, the fluctuation of the gravity center, and the constant attention of the participant during the activity because they had to catch the fish that would rise from the sea by moving on the iceberg (platform). The caught fish added points and provided direct feedback in a visual (scoreboard) and auditory way. In the second activity of gamification, participants had to get on and off the platform in time with the steps that simulate the gait pattern; in this way, participants trained static, reactive, and proactive balance. The users also received auditory and visual feedback during this activity. In addition to these feedback contributions, the scores were recorded on a designed sheet that was given to the participants after the end of each session and that they could share with other colleagues, family members, and laboratory professionals who supervised and guided the activity.

### Intervention protocol

The clinical trial, developed in laboratory conditions, was divided into eight phases:

– Phase I: Reading and signing of the Informed Consent.

– Phase II: Summoning of the participants for the initial evaluation and undertaking of the tests by physiotherapists and neuropsychologists to determine physical and cognitive abilities.

– Phase III: Collection of all the initial evaluations of each valuator and calculation of the average scores. Elaboration of a database with all the initial values of all the participants.

– Phase IV: intervention of the task using information technology in the experimental group. The total number of sessions was 16, 2 times a week, each one with a 30 min duration, after consulting previous research (Lai et al., 2013). Each session contained three series of the video game “Penguin Slide,” one series of “Step Plus” and another three series of “Penguin Slide.” After each session, the subject obtained a score that was registered in a tracking sheet of the sessions to increase the motivation of participants and their caregivers. The activity was carried out in a laboratory that contained two monitors and two video consoles so that the participants could increase adherence, motivation and improvement. In this phase, the control group does not receive sessions using the Nintendo^®^ Wii game, they continue with their usual care (memory workshops and joint mobility workshops in the center).

– Phase V: Summoning of the participants for the final evaluation and undertaking of tests (by physiotherapists and psychologists) to determine physical and cognitive abilities.

– Phase VI: Collection of the final evaluations of each evaluator and calculation of the average score. Elaboration of a database with the final scores of all participants.

– Phase VII: Elaboration of the final database, the variables blinded to the evaluators, and development of the statistical tests.

– Phase VIII: intervention of the task using the information technology in the control group once the final data was collected.

### Data collection procedure

Clinical evaluations were made by physiotherapists (MP, GC, and CS) and neuropsychologists (MV and AG) with more than 10 years’ of experience in the intervention field. These evaluations were undertaken twice: the first one after signing the Informed Consent and the second one three days later, after the end of the intervention in the experimental group, more specifically three days after finishing the last session of the treatment (16 sessions were developed in the intervention group). Once the evaluation was undertaken in the control groups, the intervention with the patients of this group took place.

A database was created using the statistical program SPSS 18.0 (available for Windows) and the Excel program to extrapolate the neuropsychological data from the E-prime computer program.

Statistical analysis was structured in four main sections:

1) Normality tests of the dependent variables were undertaken using the Shapiro-Wilk test. The description of the results of the dependent variables was also performed, using either the mean and the standard deviation or the median and interquartile range depending on the adjustment of these variables to normality.

2) Homogeneity was checked in both groups before starting treatment. For those variables that adjusted to normality, the Student’s *t*-test was used (for independent samples). If there was no homogeneity in the variables, Welch’s *t*-test was used. For those variables that suffer a deviation from normality, the Mann-Whitney U Test is used. For the gender variable, the Chi-squared statistic test was used.

3) An evaluation was performed to check if there were any significant differences in each group between the initial and final evaluations. The Student’s *t*-test was used for the parametric variables (for related samples). For the non-parametric variables, the Wilcoxon Rank-sum test was used.

4) The possibility of the results being better in one group than the other was checked. That is to say, if there was a statistically significant difference in the averages of the difference variables in the scales or tests for depression, MEC, Berg, Tinetti, Timed Up and Go Test, Oddball, ANT-ELDERLY between the experimental and control group. The Student’s *t*-test was used for independent samples in the parametric variables and the Mann-Whitney U test for the non-parametric. The effect size was calculated using the formula d = 2t/Ögl in the parametric variables and Grissom’s criteria were followed for the non-parametric ones (Dasborough, 2007). A per-protocol analysis of the effects of the applied intervention was performed. All the statistical tests were performed with a confidence trial of 95% (*p* < 0.05).

### Ethical considerations of the study

This research follows the ethical principles for medical investigations involving human subjects, in accordance with the Universal Declaration on Human Rights, General Assembly Resolution 217A III (10 December 1948) of the United Nations, the Bioethics declaration of Gijón (International Society of Bioethics [SIBI], 2000), the International Association of Bioethics (IAB), the UNESCO Universal declaration on bioethics and human rights (for Education, Sciences and Culture) and the Declaration of Helsinki of the World Medical Association, 2013 (THE WORLD MEDICAL ASSOCIATION, INC. DECLARATION OF HELSINKI Ethical Principles for Medical Research Involving Human Subjects) in its version of the 64th General Assembly, Fortaleza, Brazil, October 2013 and complemented with the Declaration of Taipei of 2016 on Ethical considerations regarding health databases and biobanks. In addition, the research project received the approval of the Experimentation Ethics Committee of the University of Seville. Before participating in the trial, each participant was informed (verbally and in writing) about the procedure and signed the Informed Consent. Also, participants give consent to the informatic processing of their data for scientific purposes, regarding legal standards. In accordance with the 15/1999 Personal Data Protection Act, the personal data that were collected from the subjects were the ones needed for the correct development of the trial. The study data will not be revealed by researchers. Participation was anonymous, although data of each participant was registered in a control list that was kept by the main researcher and only consulted when it was essential.

## Results

**FIGURE 2:**
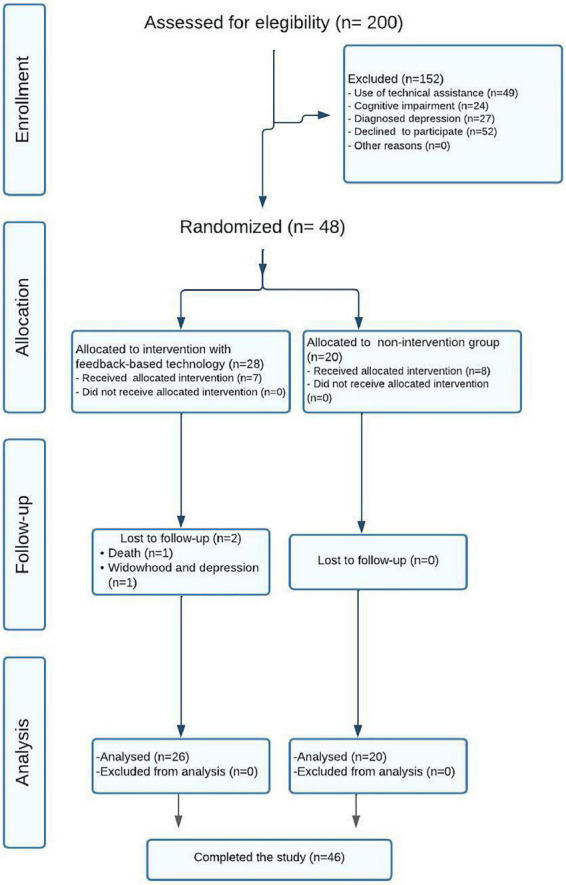
Flowchart. Consort statement.

After completing all phases of the investigation represented in the flow diagram (Figure 2), the final sample was made up by 48 users, later reduced to 46, and were organized as follows: 20 participants in the control group (40% of them were men and 60% were women) and 26 participants in the experimental group (23,1% of them were men and 76,9% of them were women). Table 1 contains descriptive values of each variable that has been analyzed, indicating the mean and standard deviation or the median and the interquartile range regarding normality tests. Table 2 the same data for the difference variables.

**TABLE 1:** Statistical descriptive values for all the study variables (*for the median and interquartile range). Own elaboration.

Variable	Group	Average	DT
Age	Control	73	7.04
	Experimental	72.19	4.79
Initial depression	Control	3.85	3.01
	Experimental	3*	5*
Final depression	Control	3.95	2.66
	Experimental	1*	1*
Initial MEC (MMSE)	Control	28.50*	3*
	Experimental	28*	2*
Final MEC (MMSE)	Control	29*	2*
	Experimental	29*	1*
Initial BERG	Control	54*	5*
	Experimental	51.65	3.11
Final BERG	Control	52.50*	5*
	Experimental	56*	3*
Initial TINETTI	Control	25.70	1.83
	Experimental	27*	3*
Final TINETTI	Control	25.60	1.95
	Experimental	28*	1*
Initial TIMED UP AND GO TEST	Control	12.16	3.21
	Experimental	11.64*	3.95*
Final TIMED UP AND GO TEST	Control	12.98*	4.17
	Experimental	8.51*	2.90*
Initial ODBALL precision	Control	99.10	0.82
	Experimental	99.5*	1.00*
Final ODDBALL precision	Control	99.50*	5.75*
	Experimental	100*	0.00*
Initial TR ODDBALL	Control	412.03	23.33
	Experimental	433.90	24.25
Final TR ODDBALL	Control	423.15	28.65
	Experimental	420.49	30.22
Initial ALERTING NETWORK effect	Control	46	52,47
	Experimental	17.86	100.73
Final ALERTING NETWORK effect	Control	5.20	65.98
	Experimental	–3.57	58.97
Initial ORIENTING NETWORK effect	Control	–6.20	35.75
	Experimental	31.57	41.58
Final ORIENTING NETWORK effect	Control	15	53.40
	Experimental	–7.71	84.99
Initial EXECUTIVE NETWORK effect	Control	67	100.93
	Experimental	73.74	52.69
Final EXECUTIVE NETWORK effect	Control	–23.20	29.62
	Experimental	15.43	75.58

*Indicates interquartile range. For this reason, in cases where the interquartile range is needed this symbol appears.

**TABLE 2:** Descriptives for the difference variables (+ for the median and interquartile range). Own elaboration.

Difference variable	Group	Average	DT
Difference – DEPRESSION	Control	0.00*	1*
	Experimental	–2.61*	2.50*
Difference-MEC (MMSE)	Control	0.00*	2.50*
	Experimental	1.0*	2.00*
Difference – BERG	Control	0.00*	1.75*
	Experimental	2.00*	4*
Difference – TINETTI	Control	–0.10	1.99
	Experimental	1.00*	2*
Difference – TIMED UP AND GO TEST	Control	1.17	2.84
	Experimental	–2.26*	2.37
Difference – ODDBALL PRECISION	Control	0.00*	5.75*
	Experimental	0.5	1.35
Difference – TR ODDBALL	Control	11.12	27.29
	Experimental	–13.40	15.59
Difference – ALERTING NETWORK	Control	–55*	37.50
	Experimental	45*	272.00*
Difference – ORIENTING NETWORK	Control	21.20	63.55
	Experimental	–39.28	77.80
Difference – EXECUTIVE NETWORK	Control	–90.20	102.80
	Experimental	–57.71	81.26

*Indicates interquartile range. For this reason, in cases where the interquartile range is needed this symbol appears.

Regarding the gender variable, there was no statistically significant difference in the distribution by gender of the patients in the groups, as χ^2^ (1.46) = 1,529, p(bilateral) = 0,216. Table 3 shows no significant differences in the initial dependent variables between groups, therefore, the groups were homogeneous at baseline.

**TABLE 3:** Statistical test to assess homogeneity between groups.

Variable	Statistical test	Significance
Age	Welch-T	0.063
Initial DEPRESSION	Mann-Whitney U	0.867
Initial MEC (MMSE)	Mann-Whitney U	0.650
Initial BERG	Mann-Whitney U	0.240
Initial TINETTI	Mann-Whitney U	0.216
Initial TIMED UP AND GO TEST	Mann-Whitney U	0.595
Initial ODDBALLL PRECISION	Mann-Whitney U	0.349
Initial TR ODDBALL	T-Student	0.07
Initial ALERTING NETWORK	T-Student	0.328
Initial ORIENTING NETWORK	T-Student	0.460
Initial EXECUTIVE NETWORK	T-Student	0.537

**FIGURE 3:**
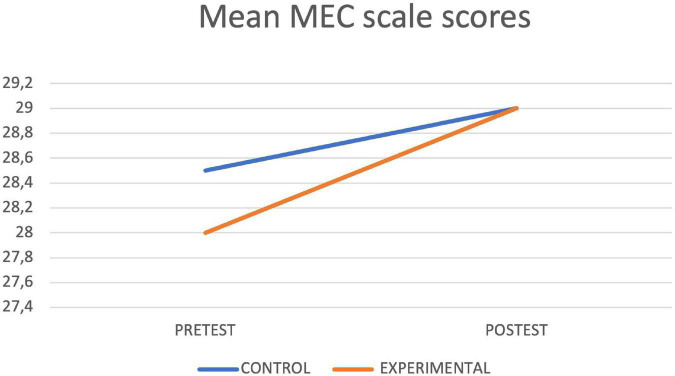
MEC lineal model.

**FIGURE 4:**
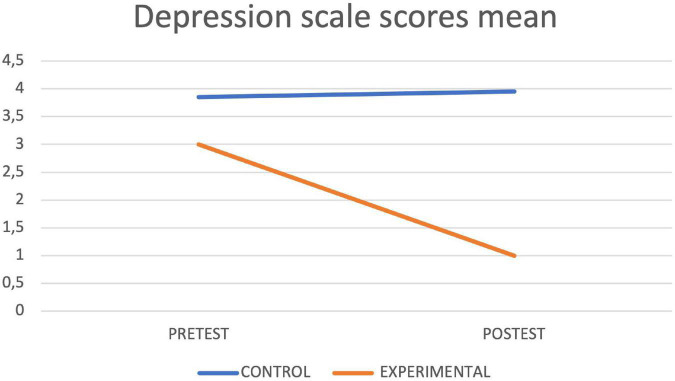
Depression lineal model.

**FIGURE 5:**
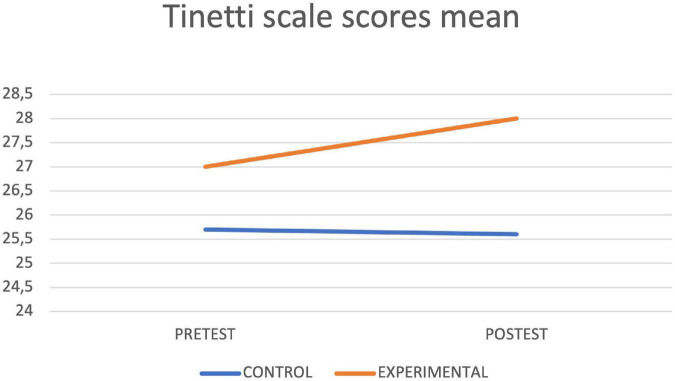
Tinetti lineal model.

**FIGURE 6:**
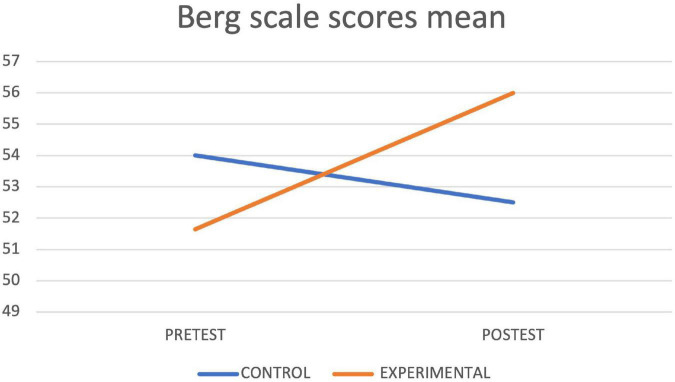
Best lineal model.

**FIGURE 7:**
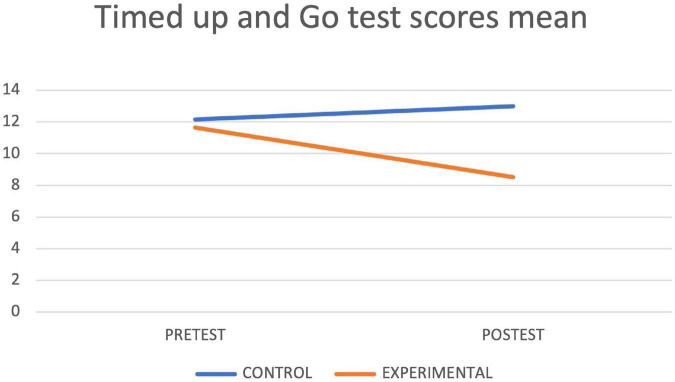
Timed up and go test lineal model.

Table 4 shows the results of the contrast tests. In the control group, an increase was observed in the seconds of execution in the final assessment in the Timed up and Go Test, while in the experimental group statistically significant differences were found between the pretest and the posttest in the values obtained in the Timed up and Go Test test, with a decrease in the seconds of execution, as well as in the Depression Scale, in the Cognitive Mini-Examination, in the Berg Scale, in the Tinetti Scale and in the average reaction times of the Oddball Test, obtaining in all cases an improvement with respect to the initial values. The graphs of marginal means are shown to illustrate the differences between both groups for the Mini Cognitive Examination, Depression, Berg, Timed up and Go Test and Tinetti scales (Figures 3–7, respectively).

**TABLE 4:** Contrast tests in the groups. Reference: own elaboration.

Variable	Group	Statistical test	Significance
DEPRESSION	Control	T-Student	0.733
	Experimental	Wilcoxon Rank-sum	0.001
MEC (MMSE)	Control	Wilcoxon Rank-sum	0.30
	Experimental	Wilcoxon Rank-sum	0.001
BERG	Control	Wilcoxon Rank-sum	0.319
	Experimental	Wilcoxon Rank-sum	0.001
TINETTI	Control	T-Student	0.825
	Experimental	Wilcoxon Rank-sum	0.001
TIMED UP AND GO TEST	Control	Wilcoxon Rank-sum	0.048
	Experimental	Wilcoxon Rank-sum	0.001
ODDBALL PRECISION	Control	Wilcoxon Rank-sum	0.305
	Experimental	Wilcoxon Rank-sum	0.082
TR ODDBALL	Control	T-Student	0.261
	Experimental	T-Student	0.003
ALERTING NETWORK	Control	T-Student	0.706
	Experimental	T-Student	0.481
COORDINATION NETWORK	Control	T-Student	0.245
	Experimental	T-Student	0.802
EXECUTIVE NETWORK	Control	T-Student	0.121
	Experimental	T-Student	0.109

Finally, an interaction between groups was executed using the difference variable (calculated through the subtraction of the obtained value in the final and initial evaluation of the main dependent variables). Table 5 shows the obtained values and the effect size (calculated following the criteria and formulas that appear in the methodology section).

**TABLE 5:** Differences in both groups. Reference: own elaboration.

Variables	Test	Unilateral significance	Effect
			size
Difference – DEPRESSION	Mann-Whitney U	0,001	0.74
Difference – MEC (MMSE)	Mann-Whitney U	0,025	0.33
Difference – BERG	Mann-Whitney U	0,001	0.837
Difference – TINETTI	Mann-Whitney U	0,0135	0.38
Difference – TIMED UP AND GO TEST	Mann-Whitney U	0,001	0.86
Difference – ODDBALL Precision	Mann-Whitney U	0,053	0.26
Difference – TR ODDBALL	T-Student	0,0025	0.886
Difference – ALERTA	Mann-Whitney U	0,5	0.76
Difference – COORDINACIÓN	T-Student	0,14	0.48
Difference – EJECUTIVA	T-Student	0,277	0.38

Significant differences were found between groups in all physical variables and only in the memory, “Difference-MEC” and “Difference-TR Oddball” cognitive variables, showing better results in the intervention group. The effect size for the physical variables was high, except for the “Difference-Tinetti” (*d* = 0.38). In the cognitive variables, the effect size was low for the memory and moderate for the “Difference-TR Oddball” (*d* = 0.886).

## Discussion

This article aimed to assess the effectiveness of using feedback-based technology on the physical and cognitive abilities of the elderly. After undertaking the clinical trial, an improvement in memory, balance, gait, autonomy, and precision speed was confirmed. The main discoveries of the study are discussed below.

### Effects of the use of technology based on feedback on cognitive and emotional capacities

Even though depression was an exclusion criterion of the study, the information obtained showed that the intervention improved depression levels in the intervention group (measured with the Yesavage Scale) and found a unilateral statistical significance in the difference-depression variable (*p* < 0,01) with an effect size of 0.74. This is promising information for clinical foresight, due to the high levels of depression amongst older adults that causes a general apathy that inevitably affects other areas of the individual (Matias et al., 2016; Lin et al., 2020). This improvement in mood could be caused by the therapeutic, fun, and motivating exercise that is practiced with technology. In addition, technology provides feedback that facilitates the learning of new strategies. This also facilitates mood improvement because subjects feel they are able to do new things, a feeling that would result in adherence to the treatment. Laver et al. (2011) compared patients that underwent conventional physiotherapy treatment and patients that underwent conventional therapy combined with treatment using the Nintendo^®^ Wii video game console at home. Users that used the device did the exercises of “Penguin Slide” and other exercises found in the strength training category. These authors concluded that users preferred a combination between conventional treatment and therapy using technology (although there was not a physiotherapist). In this way, mood improvement found in this trial could be associated with good adherence to these types of therapies. In this case, this trial has been developed with the supervision of physiotherapists that answered questions and made technology more approachable to the elderly. Participants obtained feedback on the score registers of each session, which could influence the feeling of wellbeing and mood positively, as other authors concluded (Hsu et al., 2011). In this sense, reviews on the use of exergames in adults reach the same conclusions, highlighting the decrease in the state of loneliness, the increase in social connection and a more positive attitude toward others (Li et al., 2018).

On the other hand, no discoveries were made in the depression variable, although it is an alteration of the affective and emotional field that seems to be very linked to aging (Matias et al., 2016). The criteria of the study contemplated the exclusion of people that had high scores of depression, to try to avoid the results of the attention test being covert. This fact, linked to the maintenance of their daily activities, could explain the results of this trial and the subtle improvement of memory. Although cognitive deterioration due to aging starts at 60 years old (Blanchet et al., 2018), the fact that we are referring to active older adults could be the reason why this deterioration is not noticeable. Therefore, it is essential to design therapies that focus on the prevention and promotion of healthy aging.

On the cognitive abilities, the designed task proved effective in improving memory and reaction times in the intervention group, but not in improving attention networks. Relevant results were found in the difference-MEC variable (*p* = 0,025), with an effect size of 0.33, and in Difference-TR Oddball (*p* = 0,0025) with an effect size of 0.886. Based on these results, we can say that the task partially improves cognitive abilities in the elderly. Although it has been possible to observe in other research the impact that the use of video games has on the cognitive area in institutionalized patients (Jahouh et al., 2021), no previous study was found that uses the Oddball and Attention Network Test as an initial evaluation for a therapeutic program using the Nintendo^®^ Wii video game console, because most studies that use this device focus on improving physical abilities (Clark and Kraemer, 2009; Hsu et al., 2011; Young et al., 2011; Reed-Jones et al., 2012), on preventing falls (Williams et al., 2010; Young et al., 2011; Lai et al., 2013), on the treatment of neurological pathologies (Saposnik et al., 2010; Mendes et al., 2012; Pompeu et al., 2012) or on the recall of the protocol of the treatment once time has passed after the intervention (Pompeu et al., 2012). Results cannot be compared, although it can be argued that there is an improvement in cognitive functions through their connection with physical abilities. Moreover, there is a study developed by Persad et al. (2008) where cognitive and physical functions are linked, determining that cognitive dysfunctions could be predictive of fall risk due to impairment in balance and gait. Also, the research undertaken by Scherder et al. (2007), where there is a close link between gait and cognition, and remarks the importance of continuing to investigate in this field. Atkinson et al. (2007) also analyzed the interaction between specific physical and cognitive variables and came to the conclusion that global cognitive functions and executive functions predict the decline in gait speed, although this relation can be attenuated by comorbidity factors (depression above all). If the task using feedback-based technology improves physical skills (gait speed above all, assessed with the Timed Up and Go Test), it can also improve global cognitive skills due to their (studied already by other authors). Nevertheless, it cannot be said that this partial improvement in the cognitive functions is the result of performing this activity, of the improvement of the physical capacities, the mood improvement, or the sum of it all. Even so, the use of a video game console enables performing a physical task and having cognitive stimulation at the same time, because the user needs to pay attention to the aim of the exercise, has positive feedback (the score they obtain), and has visual and auditory stimuli that guide them when they have to get on or off the platform. It allows the subject to work on alertness, orientation and planning skills and memory, apart from physical skills. In this sense, we can consider that the use of feedback-based technology activates different neural circuits, obtaining clinical benefits: low cost, adherence to the treatment, fun and motivating aspect, the possibility of creating intergenerational bonds, and the feasibility of being used at home.

Regarding attention networks in this group, there were no statistically significant findings in the undertaking of the activity. Attention networks engage complex neural circuits that are already impaired because of aging. Two months is not enough to activate the mechanisms of neuroplasticity that could change the results of the Attention Network Test; for this reason, the monitoring for a longer period is set as foresight. The precision of patients in the test was low, resulting in the feeling that the task was difficult for them and that the tips had not been useful, causing omissions in the task on many occasions. This information suggests that the device has to be reviewed and improved to facilitate its use in this age group and that the results related to the attention networks need to be analyzed with caution. Even so, it is worth highlighting that in the experimental group, there is a decrease in the effect of alertness and an increase in the effect of orientation, which happens to be the same as the findings of Mahoney et al. (2010) (even though it is not significant). It would have been interesting to include blood pressure levels because this group of researchers tries to associate low blood pressure and a decline in the executive network. The discoveries of their research proved that the decline of executive function in older adults and lower blood pressure in the frontal lobe could be responsible for the differences in attention regarding the different attention requirements.

With respect to the control group, our findings in attention were not significant, however, the descriptive ones in the Oddball test showed an increase in reaction time and a decrease in the percentage of accuracy. This information is the same as other authors’ studies that state aging is associated with cognitive decline and that attention alterations affect the other cognitive functions (Nóbrega-Sousa et al., 2020). Concerning attention networks (alerting, orienting, executive), there was a decrease in their effect in the control group that was not significant. This fact differs from the discoveries of Jennings et al. (2007), who found less alertness in the assessed group of older adults and did not find differences in the orientation and executive network. Despite this group of researchers basing their study on the Posner paradigm, the designed routine differs from the one used in the study. The same happens with the experiment by Gamboz et al. (2010), who used a different routine and came to the same conclusions as Jennings et al. (2007). Nonetheless, discoveries agree with Zhou and collaborators on the decline of the executive network (Zhou et al., 2011), without finding any alterations in the alerting or the orienting network. Comparing the results with the ones of Marrufo and collaborators’ research (Vazquez-Marrufo et al., 2011), they are not similar regarding the effect on the networks. However, the average values of the trials were considered in this research without dividing the instrument into blocks. These studies compare the attention networks of older adults and young adults in one measurement. Still, they do not assess (with therapies or without them) how the attention networks can change in the same subject, an aspect that is included in this study. Attention networks tend to worsen, as in other studies that confirm that there is impairment in attention, executive function, memory, language, and visual-spatial processing as a consequence of aging (Drag and Bieliauskas, 2010). However, as a foresight, it could be interesting to improve this instrument, because it could be a tool that determines if the functioning of the networks is either a sign of normal or pathological aging, as shown in their review (Mcdonough et al., 2019). This is an important aspect to focus on the prevention or delay of possible pathologies associated with aging and on the promotion of healthy aging.

For the improvement of the tool, the discoveries of other research by Mahoney et al. (2012) could be of special interest, where a trial was designed to determine how many participants (young people and adults) could benefit from receiving multisensorial cues of alertness and orientation. The results of both groups showed effects of orientation for auditory-somatosensory, audiovisual, and visual-somatosensory stimuli. After analyzing the group separately, they came to the conclusion that young people had more advantages for reaction times in the auditory-somatosensory cues and the older adults had more advantages for the audiovisual cues. For this reason, it could be useful to implement an auditory cue to the Attention Network Test-ELDERLY to improve its effectiveness, regarding the information provided in this study.

### Effects of using feedback-based technology on physical abilities

Regarding physical abilities, the task was effective to improve balance and gait in the intervention group, due to the finding of unilateral statistical significances in the difference-Berg variable (*p* < 0,01) with an effect size of 0.827; in the difference-Tinetti variable (*p* = 0,0135) with an effect size of 0.38 and in the difference-Timed up and Go Test variable (*p* < 0,01) with an effect size of 0.86. This data happens to be the same as in previous research by other authors (Williams et al., 2010), who found significant improvements in the Berg scale, especially in anteroposterior and mediolateral balance (with both open and closed eyes). Other research found improvements in balance and gait (Nitz et al., 2010; Toulotte et al., 2012). Other researchers (Clark and Kraemer, 2009) stated that the Wii Balance Board was similar to stability platforms and that it could be a good alternative because they were cheaper and it is easy to use, even at home. Moreover, there are similarities with other studies (that focus on fall prevention) in the data obtained for this variable, highlighting the Lai and collaborators’ research (Lai et al., 2013), which used a protocol that has been a reference for this essay, more specifically for the design of the intervention.

It is possible that the exposure to auditory and visual stimuli through the video game justifies the improvement of postural stability and balance, as other authors have already discussed (Hatzitaki et al., 2009; Dozza et al., 2011). This exposure can also diminish the incidence of falls, causing an increase in the autonomy of older adults. One of the main aims of the gerontological field is the prevention of falls because they are considered one of the main causes of disability in this age group. We can confirm that 1/3 of people over 65 years old fall at least once a year and half of them fall a second time (Tinetti, 2003). The experimental group of this trial reached an absence of any risk of falling according to the Timed up and Go Test. These data agree with the results found in the research undertaken by Schoene et al. (2013). Among their results, it is worth highlighting the significant difference between reaction time and the improvement of postural balance and cognition. In this case, the experimental group had the best results. These authors argue that the ceiling effect in certain subjects could be caused by good functionality before the start of the treatment; the same can have happened in this trial.

As mentioned in the results, in the control group, a significant difference was found in gait (measured with the Timed up and Go test). And even though there were no significant differences in the physical examinations, from a clinical point of view, the worsening of the results due to the passage of time is considered relevant. The significance obtained in only two and a half months was very noticeable. There is a possibility that the increase in temperatures during the final evaluation (that took place in May) could be responsible for the reduction in the activity of the participants that influenced the gait speed. These findings confirm the idea that the process of aging affects balance and gait, causing people to become more insecure about their movement and more susceptible to falls and dependence (Alexander, 1996; Dziechciaż and Filip, 2014). Physical changes are more noticeable inpatients over 80, which could be the reason for the absence of significance on balance. Other reasons could be the inclusion criteria that require independence; the time of the established protocol, and the preservation of the stimulation and mobility activities developed in the center of origin of the participants (usual care).

However, this study has maintained the usual care in both groups (memory and joint mobility workshops) and has only applied technology based on feedback as a tool to optimize physical and cognitive abilities. As a future perspective, it could be interesting to join the proposed protocol using the Nintendo ^®^ Wii video console, conventional physiotherapy exercises and cognitive stimulation, as other authors have done in previous research (Waliño-Paniagua et al., 2019; Cano-Mañas et al., 2020). Likewise, we could create diverse groups with technology based on different feedback, to check if the benefits are found in all possible devices or in some more than in others.

In conclusion, the development of this study has shown that the use of the protocol on older people improves memory, precision speed, balance, gait, global autonomy and mood. Although there are previous investigations that have been able to obtain these same findings separately (Li et al., 2018; Perrochon et al., 2019; Jahouh et al., 2021; Liu et al., 2022), our study is proposed as an integrative strategy that can holistically address all the areas that make up the individual, maintaining their functionality and capabilities. In addition, unlike other studies, the incorporation of instruments that assess attention as a complex cognitive function, once the improvements we propose have been added, could be effective in anticipating the choice of the best therapy for the participants, since we could acquire data on the way the brain processes and this could make it easier for us to choose the game that best suits each need. Likewise, the findings in the control group highlight the consequences that the aging process can cause on the physical, cognitive and emotional spheres, and the use of technology based on feedback may be an effective preventive strategy to maintain functionality and avoid dependency.

## Data availability statement

The raw data supporting the conclusions of this article will be made available by the authors, without undue reservation.

## Ethics statement

The studies involving human participants were reviewed and approved by Ethics Committee of Experimentation of the University of Seville. The patients/participants provided their written informed consent to participate in this study.

## Author contributions

M-LB-L conceptualized the idea. M-LB-L, GC-M, and CS-S designed the study and wrote the manuscript. MV-M, AG-C, CS-S, and GC-M collected the data. M-LB-L, MV-M, and CS-S analyzed the data. All authors contributed to the final version and approved the final manuscript for publication.
